# Supplementary material for: Muricholic Acids Promote Resistance to Hypercholesterolemia in Cholesterol-Fed Mice
Source: Int J Mol Sci. 2021 Jul 2;22(13):7163. doi: 10.3390/ijms22137163 (PMC8269105; doi:10.3390/ijms22137163)
Supplement: Supplementary file 1 [file ijms-22-07163-s001.zip › ijms-1257699-supplementary.pdf]

Figure S1

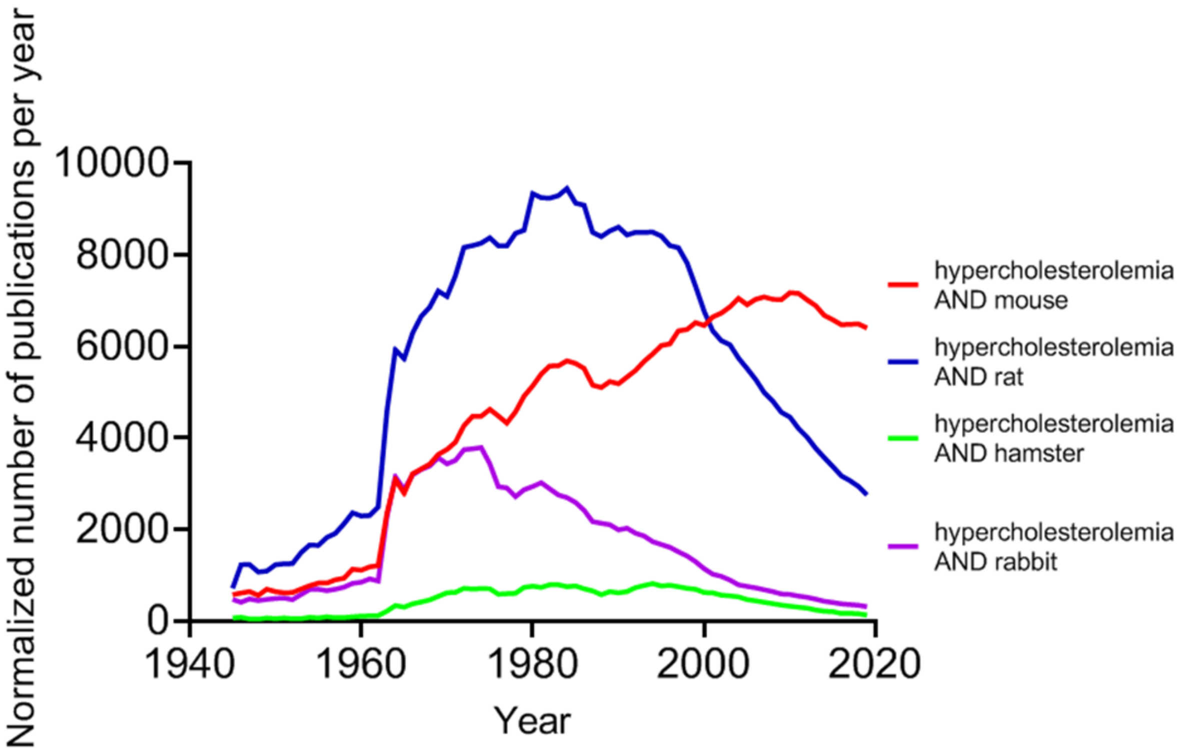

Figure S1. Bibliometric analysis of PubMed Indexed publications on hypercholesterolemia in common laboratory animals.

Normalized number of publications per 100000 publications per year from PubMed searches(https://esperr.github.io/pubmed-by-year/)

Figure S2

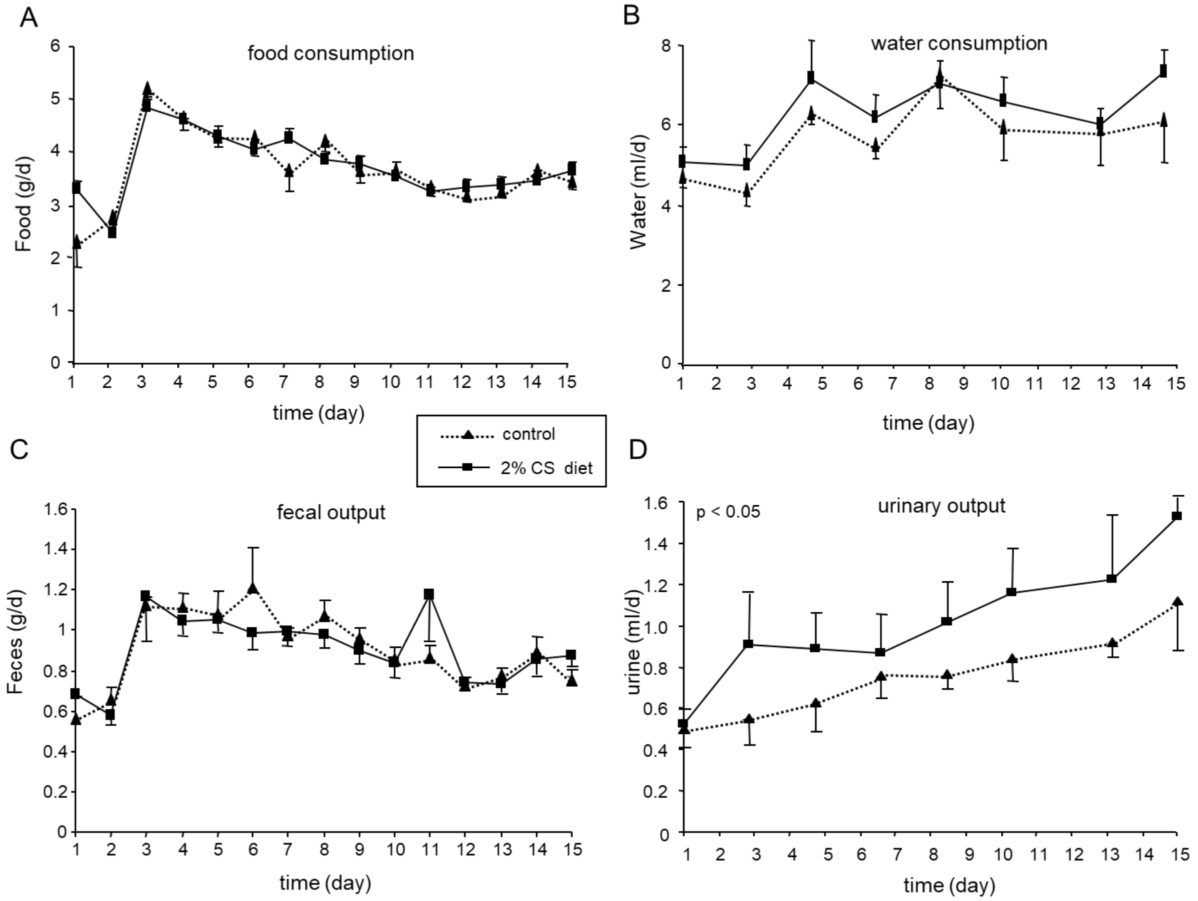

Figure S2. Assessment of food and water consumption along with fecal and urinary outputs in cholesterol-fed mice.

8 weeks-old mice fed a standard diet (control) or a 2% cholesterol-enriched diet for 15 days (2% CS) were housed in metabolic cages. (A) Food and (B) water intake were monitored daily as well as (C) fecal and (D) urinary outputs. Graphs represent mean ± s.e.m (n=8 per group). *$P<0.05$* determined by ANOVA2 with repeated measures.

Figure S3

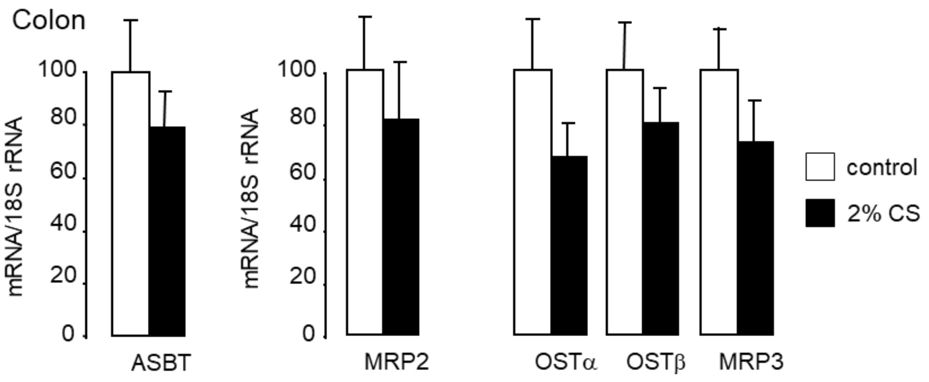

Figure S3. Gene expression of colon bile acid transporters in cholesterol-fed mice.
mRNA levels of bile acid transporters in the colon of standard diet (control) or 2% cholesterol-fed (2% CS) mice. Bar graphs represent mean ± s.e.m (n=8 per group).

Table S1. Composition of liver bile acid pool in control and cholesterol-fed mice.

Mean ± s.e.m (n=8 per group). *P* value were determined by Student's t-test with Welch's correction.

		Intrahepatic concentration (ng/mg)		*T-test*	Percentage (%)		*T-test*
	Heuman's Hydrophobicity Index (HI)	Ctrl	2% CS	*P value*	Ctrl	2% CS	*P value*
LCA	+1	1.07 ± 0.18	1.21 ± 0.17	*0.59*	1.20 ± 0.26	1.01 ± 0.11	*0.49*
DCA	+ 0,59	2.10 ± 0.99	3.22 ± 0.71	*0.37*	2.06 ± 0.88	2.66 ± 0.6	*0.58*
CDCA	+0,46	4.84 ± 0.95	5.81 ± 0.48	*0.36*	5.03 ± 0.68	4.90 ± 0.3	*0.86*
CA	0	41.13 ± 6.4	34.34 ± 3.55	*0.36*	**43.64 ± 5.01**	**29.07 ± 2.75**	***0.025***
HDCA	-0,35	1.23 ± 0.14	1.15 ± 0.28	*0.81*	1.35 ± 0.2	0.93 ± 0.18	*0.16*
β-MCA	-0,78	**33.26 ± 4.96**	**62.98 ± 4.69**	***0.002***	**36.40 ± 5.52**	**53.23 ± 3.45**	***0.025***
α-MCA	-0,84	9.52 ± 1.03	9.70 ± 1.47	*0.92*	10.19 ± 0.93	8.08 ± 0.99	*0.16*
Primary BA	/	**88.85 ± 5.53**	**115.16 ± 4.97**	***0.009***	95.38 ± 0.74	95.17 ± 0.80	*0.99*
Secondary BA	/	4.40 ± 0.92	5.97 ± 1.04	*0.41*	4.62± 0.74	4.83± 0.80	*0.99*
Primary BA/Secondary BA	/	22,8 ± 3,49	24,75 ± 6,73	*0,750*	/	/	/
Total	/	**93.25 ± 6.15**	**118.55 ± 5.66**	***0.014***	100	100	/

Table S2. Composition of fecal bile acid pool in control and cholesterol-fed mice.

Mean ± s.e.m (n=8 per group). *P* value were determined by Student's t-test with Welch's correction.

		Fecal excretion (µg/days)		*T-test*	Percentage (%)		*T-test*
	Heuman's Hydrophobicity Index (HI)	Ctrl	2% CS	*P value*	Ctrl	2% CS	*P value*
LCA	+1	**9.44 ± 0.46**	**25.38 ± 5.11**	***0.01***	3.77 ± 0.22	4.47 ± 0.74	*0.4*
DCA	+ 0,59	**174.94 ± 13.84**	**295.11 ± 29.33**	***0.004***	**68.61 ± 1.2**	**53.35 ± 2.58**	***0.0003***
CDCA	+0,46	**4.41 ± 0.38**	**10.04 ± 2.281**	***0.03***	1.74 ± 0.1	1.73 ± 0.35	*0.9*
CA	0	**14.48 ± 1.82**	**50.02 ± 16.15**	***0.05***	5.64 ± 0.45	8.92 ± 2.83	*0.3*
HDCA	-0,35	**2.97 ± 0.18**	**8.52 ± 1.89**	***0.01***	1.19 ± 0.11	1.48 ± 0.25	*0.3*
β-MCA	-0,78	**36.11 ± 3.17**	**110.77 ± 7.96**	***$0.55x10^{-5}$***	**14.21 ± 0.97**	**20.39 ± 1.43**	***0.005***
α-MCA	-0,84	**12.29 ± 0.9**	**54.86 ± 7.88**	***0.0003***	**4.83 ± 0.11**	**9.65 ± 0.88**	***0.0003***
Primary BA	/	**67.3 ± 5.62**	**225.7 ± 24.2**	***$0.8x10^{-4}$***	**26.43 ± 1.25**	**40.69 ± 3.03**	***0.001***
Secondary BA	/	**187.35 ± 14.14**	**329.01 ± 34.58**	***0.003***	**73.57 ± 1.25**	**59.31 ± 3.03**	***0.001***
Primary BA/Secondary BA	/	**0.36 ± 0.02**	**0.71 ± 0.1**	***0,008***	/	/	/
Total	/	**254.65 ± 18.74**	**554.71 ± 48.14**	***0.0002***	100	100	/
